# Development of a Mobile App for Clinical Research: Challenges and Implications for Investigators

**DOI:** 10.2196/32244

**Published:** 2022-04-01

**Authors:** Shibani Chettri, Vivian Wang, Eli Asher Balkin, Michael F Rayo, Clara N Lee

**Affiliations:** 1 College of Public Health The Ohio State University Columbus, OH United States; 2 College of Medicine Central Michigan University Mount Pleasant, MI United States; 3 Department of Integrated Systems Engineering The Ohio State University Columbus, OH United States; 4 Department of Plastic and Reconstructive Surgery College of Medicine The Ohio State University Columbus, OH United States; 5 Division of Health Services Management and Policy College of Public Health The Ohio State University Columbus, OH United States; 6 The James Comprehensive Cancer Center-Solove Research Institute The Ohio State University Columbus, OH United States

**Keywords:** mHealth, mobile app, patient-collected data, data security, mobile health, patient data, clinical research, research facilities

## Abstract

Advances in mobile app technologies offer opportunities for researchers to feasibly collect a large amount of patient data that were previously inaccessible through traditional clinical research methods. Collection of data via mobile devices allows for several advantages, such as the ability to continuously gather data outside of research facilities and produce a greater quantity of data, making these data much more valuable to researchers. Health services research is increasingly incorporating mobile health (mHealth), but collecting these data in current research institutions is not without its challenges. Our paper uses a specific example to depict specific challenges of mHealth research and provides recommendations for investigators looking to incorporate digital app technologies and patient-collected digital data into their studies. Our experience describes how clinical researchers should be prepared to work with variable software and mobile app development timelines; research institutions that are interested in participating in mHealth research need to invest in supporting information technology infrastructures in order to be a part of the growing field of mHealth and gain access to valuable patient-collected data.

## Introduction

The rapid adoption of internet-connected digital devices is facilitating a change in how health care providers can monitor patient health. The proliferation of devices, such as cellular phones, tablet computers, and smartwatches, permits clinicians to gather data in ways that were previously impossible. Not only do these devices produce more data, but they also produce more useful data since they can be collected while patients are attending to their daily lives.

As more mobile technology and devices become available, health services research is also evolving [1,2]. Research on the delivery of health care no longer needs to restrict data collection to health care facilities or to the timing of clinical schedules. This proliferation of new research tools has also dramatically changed the roles and skills required of modern clinical and health services researchers. Such investigators must become accustomed to research with a substantial medical informatics component.

This paper describes the challenges that mobile health (mHealth) research can pose for clinical researchers, including unique issues with investigator roles, patients as researchers, data security, contracting with app developers, and informatics infrastructure. We use the example of a specific research study to illustrate these challenges and provide recommendations.

## The Rise of mHealth

Three technological trends have facilitated the shift toward mHealth: daily carry, reliable connectivity, and near-universal usage [3,4]. The consistent use of cell phones, tablets, and smartwatches by people throughout the day for web browsing, email, music, calls, and navigation means that these devices are in close proximity to potential research participants for most of the day. Thus, health care research can be conducted using data collected by these devices, no longer requiring patients to carry additional devices or modify their daily routines [5-8].

Predictable internet connectivity allows devices to automatically send patient data to health care providers and researchers without the need for patient intervention. This reduces the burden of participation for the patient, while permitting providers to review the data in preparation for the patient’s next scheduled appointment. Medical staff can also monitor the patient from afar and contact the patient for intervention if necessary.

The nearly universal use of internet-connected devices, especially cell phones, makes population-based data collection more feasible, which has important implications for health equity. Specifically, populations that have been left out of prior research may become more accessible. Finally, it also opens up possibilities for dissemination and implementation of interventions developed using mHealth, thus facilitating a wider experience of benefits at a lowered preparatory and operational cost.

## WORDS: An Illustration

The Women and Oncologists Reaching Decisions about Surgery (WORDS) study sought to understand how women with breast cancer make decisions about surgery, and how they communicate with their clinicians about treatment options. Study participants recorded their conversations with surgical oncology, radiation oncology, and plastic surgery clinicians using RecorDr, a smartphone app we created. Because of the multiple patient-provider conversations across multiple locations that is typical in determining the optimal care plans for these patients [9], our patient-as-researcher method would be particularly valuable.

The primary outcomes of the WORDS study were (1) to develop and test a mobile app in a clinical setting for research and (2) to evaluate communication and decision-making about contralateral prophylactic mastectomy. The WORDS study had a total of 105 participants. A control group was not used.

The benefit of using our mobile app was that data collection would not need a research assistant at each appointment. In effect, the patients were deputized as research assistants, responsible for recording their encounters with physicians. They went through a brief orientation during their first visit to the cancer center, but then they could make additional recordings at other visits, which facilitated data collection at dates, times, and locations where the formal research team could not participate. The app also enabled patients to record conversations with family members if they chose to do so. Such broad data collection overcomes the prior obstacles of many communication studies, provides a more complete picture of the decision-making process, and increases the generalizability of research to nonacademic settings.

In order to deputize these patients and realize these benefits, our study required a substantial informatics component. However, most clinical and health services researchers do not have informatics expertise [10]. This highlights the importance of team science and collaborating with investigators who provide that expertise. Even then, the principal investigator (PI) must be familiar enough with the particular area to communicate effectively across the assembled disciplines, including software design, software development, data infrastructure, and data security.

Within our study there were three main teams: the clinical research team, the systems engineering team, and the information technology (IT) team. The PI was a decision scientist, health services researcher, and surgeon, with expertise in decision-making about breast cancer treatments. The clinical team provided the clinical context for the app design and oversaw the patient and clinician recruitment procedures. The engineering team oversaw the app design, app development, and data transfer procedures. The clinical team relied on the engineering team and the developer. The clinical and engineering teams entrusted the medical center’s IT department to develop the infrastructure and procedures for secure data transfer and storage.

## Investigator Roles

As investigators begin to use new technological developments, they face a foundational choice between acquiring the new skills themselves or collaborating with a larger research team comprising experts in the required disciplines. In mHealth, the former option of learning about computer programming, mobile app development, and digital data storage is not highly feasible for most clinical researchers. The latter option, the more common choice and the hallmark of modern team-based science, requires research collaborators with the required expertise and willingness to collaborate. In the case of mHealth programs, collaborators often hail from such fields as engineering, computer science, and informatics. Clinical investigators doing this type of research, however, should acquire enough basic knowledge about app development and informatics to collaborate meaningfully with these parties.

We recommend that academic centers engaging in mHealth research have “common-pool” resources available for all researchers to use, such as in-house methodological consulting to aid in the development of practices and procedures for the use of mHealth technologies. The mHealth shared resource at the Stephenson Cancer Center at the University of Oklahoma is an example of a common-pool site that houses a variety of important mHealth technologies as well as connections to clinical informatics experts [11]. Additionally, short courses for researchers on contracting and working with programmers would aid in the process.

Institutions with infrastructure to support mHealth research will reduce the amount of time spent by research teams figuring out who in the organization is properly positioned to offer the services needed, or at least to inform the team that those services could not be offered. Providing clarity to researchers regarding which support services are currently available and how to access them would increase the development and adoption of mHealth research tools. This would allow research teams to know early on whether a project is not feasible within the existing infrastructure, thus saving them time and resources. In our experience, although our research project was approved to take place at our institution, we only found out several months later that some of the necessary components of our study, such as a secure data storage system and developers who had familiarity with mobile app data security, were not readily available to our research team through our university. We then spent a significant amount of time looking into third parties who provided these services. We suggest that researchers familiarize themselves with the mHealth resources at their institutions and consider the availability of an internal mobile app developer who is familiar with mHealth research, a dedicated IT department that is familiar with the needs of mHealth researchers, and the mobile data security measures currently in place at the institution. A successful model offers these services at low or no cost to research teams. Universities that choose to invest in integrated clinical informatics centers will be better equipped to keep up with the fast-paced changes in mHealth. Research and researchers who inquire about these services will be better able to anticipate the logistics, hurdles, and feasibility of their mHealth projects.

## Participants as Deputized Researchers: New Challenges

The involvement of research participants as data collectors creates new challenges. These new initiates will generally have far less training than a typical research assistant, will not be under the employ of the research team, and will generally need some short- or long-term benefit for participating [12]. Usability issues regarding study tools that a typical research team could easily address can completely derail these new, decentralized teams. We conducted two rounds of informal, formative usability testing before our first app release, and we had to quickly revise the app after the first release after new usability issues were revealed. The team had to be constantly vigilant, as the participants would not necessarily signal when they could not or would not complete their tasks; instead, they often dropped out. We provided financial reimbursement to participants, but only at the point of enrollment rather than after each data collection point. We also provided a nonfinancial incentive to mitigate participant dropout, allowing participants to keep their recordings and including a bookmark feature. This feature enabled them to tap their screen whenever something occurred that they would want to refer to later. In this way, their work as a researcher was serving their needs as a patient.

We suggest that when conducting research that includes participants as data collectors, researchers should minimize the burden for participants. If feasible, researchers should schedule financial reimbursements after the completion of different stages of data collection (eg, after successful recording). Apps should be designed to provide a nonfinancial benefit to aid in participation and retention. Additionally, usability testing in focus groups that closely resemble the demographics of the target population of the study will reduce the need for large revisions after the release of the mobile app. Although comfort with mobile technology may vary across demographics, participants who use mobile internet data technologies will likely also be comfortable with using mobile technologies in mHealth studies [13]. Clinical researchers should also consider simulating the characteristics of the setting in which the app will be used and take into account factors such as time constraints of clinical schedules, participants being in an unfamiliar setting, and needing to connect to a different Wi-Fi network than usual. Teams should also be prepared to conduct usability testing in the actual study setting that is as similar as possible to the actual setting, such as in clinic and with actual patients who are receiving care rather than just with cancer survivors who have completed treatment or healthy volunteers. Additionally, researchers should touch base with participants throughout the project to get feedback about which technological processes are working and which are problematic, so that problems can be addressed in real time. We tracked participant uploading of recordings to the server, and we suggest such monitoring after the launch of the app in order to incorporate an automatic notification system to assess for usability issues. Such feedback could be obtained via targeted inquiries in the app itself, using metrics such as number of data files received, and less time spent on the app. The app itself could then target users who may be using the app less frequently or are encountering usability problems. Other feedback options include surveys, phone calls, or in-person inquiries by on-site research personnel. However, such approaches add burden to study participants, when a major goal of mHealth research is to minimize participant burden and research staff involvement.

## Data Security in mHealth

### Overview

As mHealth grows, researchers will have to navigate data security concerns for more types of data and a wider range of situations. Since there are few industry-wide standards, each project and institution may need to develop its own approach.

### The Health Insurance Portability and Accountability Act

Questions are arising about what constitutes protected health information (PHI) under the Health Insurance Portability and Accountability Act. Initially, the Institutional Review Board (IRB) approved our study as not involving PHI. They considered the voice recordings not identifiable. Thus, we considered commercially available data storage options, such as Amazon Web Services (AWS) and Dropbox, as well as university-managed options, such as Enterprise Box and the internally owned and operated medical center storage server. After taking into consideration cost, reliability, and security, we selected Enterprise Box, which was already implemented in our university and medical center. However, while the project was underway, the university and medical center security offices changed their interpretation of the institutional data classification standards, deeming voice data as PHI.

This change, coupled with security concerns of nonuniversity devices (ie, the mobile devices of our patients), meant that a wholly new data storage solution had to be designed and implemented. The solution consisted of a new secure medical center server for storage and secure file transfer protocol (SFTP) for data transfer from the app to the server. Setting up the server and SFTP involved a partnership between our team, the medical center IT department, and the university and medical center security office. Each of these steps, including evaluating the problem (ie, audio recordings as PHI), identifying solutions (ie, server and SFTP), and implementing the solution (ie, setup of the server and SFTP) took several months, with the final implementation completed 1 year after the storage problem was identified.

Ultimately, three important data management lessons emerged regarding data transfer, data storage, and participant data security.

### Data Transfer

Data collected on a mobile app need to be vetted before being transferred to an institutional server. Additionally, data collected on personal patient devices need to be screened, since the recordings are made outside the jurisdiction of the medical center. We sent all files from mobile devices by SFTP to a server environment controlled by our institution but outside of the firewall (ie, a DMZ [demilitarized zone] network), removing the ability of malicious files or actors to infiltrate the secured institutional network. Only specific processes on specific ports within the secured network were allowed access to the file system in the DMZ network. These processes periodically moved the patient conversation audio files between the DMZ network and the secured network.

### Data Storage

When selecting a modality for PHI storage, researchers need to consider the security level of their storage system. Commercially available storage options, such as AWS, Dropbox, and Box, may not meet the institutional or regulatory security standards to house PHI; thus, other options need to be used. As new forms of patient data continue to emerge, institutional classifications of these new data may not yet be determined at academic centers. We recommend that teams encountering similar uncertainty regarding patient data classification handle patient data as PHI to prevent the need to change security standards after projects are active.

### Participant Data Security

By collecting data on portable, user-controlled devices, the data are only as secure as the device itself. Recording data are vulnerable since patients have all their recordings on their smartphones. If a patient loses their phone, recordings could be accessed by others. Researchers should be prepared to train patients in security hygiene, such as making sure mobile devices are password protected and being aware of where they leave their devices. Additional considerations to minimize privacy and security threats include training participants to be familiar with the type of data being collected and the use of the collected data. Patients should also be reminded to prohibit data access over unsecure Wi-Fi networks or hot spots [14]. Although researchers can take these precautions, a data breach on the patient’s personal device remains a risk, over which investigators have limited control.

## Contracting With an Independent App Developer

Much of mHealth research requires coordination between the core research team and an app developer. Most app developers are accustomed to creating apps on a contractual basis for monetary compensation and not necessarily for research. They may be unfamiliar with the flow and pace of clinical research and the importance of flexibility, especially when working with a protocol office, IRB, or medical center information security team. App development for clinical research requires stringent data security and privacy provisions to ensure that patient data are protected. We identified a professional developer through our professional network and negotiated a contract based on what we thought would be needed. However, requirements of the medical center IT department and the IRB required multiple changes and increasing complexity to the app. We found that our first developer was not accustomed to this level of complexity and could not accommodate multiple changes. We changed developers, which resulted in delays associated with hiring and onboarding a new developer. Our second developer tried to build off of the existing app for several months but eventually concluded that building from scratch would be more effective. The second developer was a computer science student who had the necessary skills and flexibility for the project.

We recommend that institutions have their own app developers with mHealth research experience, and that they create infrastructure to support hiring of external app developers when needed. The mHealth center at the University of Pennsylvania offers such support by searching for and preapproving app developers on behalf of research teams qualified to take on clinical projects with a strong informatics component. This saves the research team valuable time and resources [15]. When selecting a mobile app developer, researchers should seek those with experience related to research and implementation, as opposed to creating proofs of concept or demos only. For future studies, our goal is to design a team that is composed of specialists in app development, user interface and user experience design, and security. While it would be preferable for the developer to have, or be trained to have, sufficient understanding of security and other relevant areas, this may be difficult to find. Instead, we suggest looking for developers with experience building iOS and Android natively, as opposed to using libraries’ cross-platform software. They will be more likely to be able to create apps that are fit to transfer large data files quickly and securely. Additionally, it is crucial to make clear the scale of the app and the expectation for the end product app. We also recommend that investigators consider the levels of expertise, skill, and flexibility over the level of formal education.

## Conducting Research Within an Evolving IT Infrastructure

mHealth research may require working within an IT infrastructure that was not designed to support research. At many medical centers, IT staff members are more familiar with the clinical operational IT needs of the medical center, rather than with informatics research. Medical center IT departments may assign research projects lower priority than urgent, daily clinical IT needs. For our study, our institution did not have a standardized protocol for informatics research at the time. We were not assigned one person from the IT department to be dedicated to our project, leaving no clear point of contact or consistent approach to making progress. For example, when we sought to develop a firewalled storage solution for our data, we had challenges in clearly communicating this need and determining which types of expertise were required. We depended on our IT collaborators to identify the right types of expertise required. However, they were not necessarily familiar with the urgency of the project, which was related to the award period of the study’s funding; the usability of the mobile app in the clinic; or security issues for audio PHI from patient mobile devices.

Currently, many universities do not have the resources and infrastructure necessary to keep up with the demand for informatics integration into clinical research [16,17]. We contacted multiple institutions and found only a few that had mHealth-related development cores. Most institutions reported that they rely on medical center IT and IT security to help with server-side issues and contract out software development to third-party companies. However, informatics plays an integral part in advancing modern research, and its contribution will only grow in the future. Retail and service-oriented industries are using smartphones and other technology to tailor their products and accessibility to their customers. Similarly, technology could be leveraged to not only gather data from patients but empower patients to be data collectors in their own right, thereby potentially enhancing their experience in health care settings while also improving the quality of the data received.

We recommend that institutions develop informatics infrastructure specific to mHealth research. This would include technologies such as on-site mHealth experts and support personnel, standardized mHealth procedures, and improved data security infrastructure. A designated department should be established for mHealth and IT-dependent research that is distinct from IT for the clinical enterprise. A designated department will streamline projects by housing all mobile app research experts in one place and benefit from lessons learned across projects. Our institution now has a research IT department, which is developing infrastructure and standardized procedures. The University of North Carolina at Chapel Hill has the Connected Health for Applications and Interventions Core, which serves as a centralized support center for investigators seeking to incorporate mobile technology, user inquiry, and graphic design into their research [18]. Similarly, Duke University’s Mobile App Gateway is a “one-stop shop” for assistance in the development of research-focused mobile apps [19]. It offers assistance with locating and contacting the appropriate IT staff, support with external vendors, contracting, and preapproved app-related language for IRB submissions [19]. It offers introductory design training workshops for researchers interested in using mHealth technologies, but who do not have a background in digital design.

## Interactions in a Complex System

**Figure 1:**
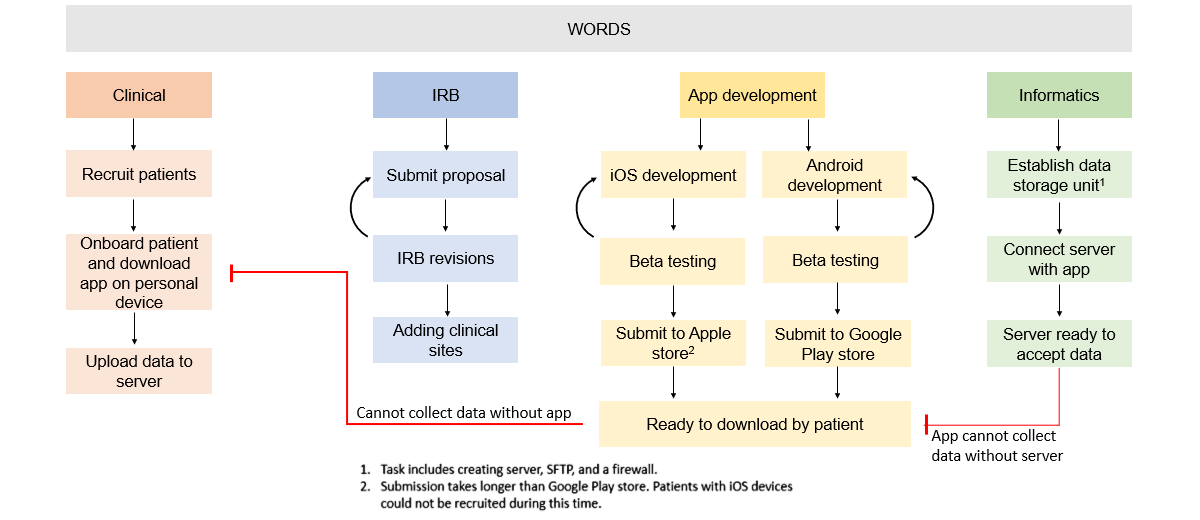
Project workflows. IRB: Institutional Review Board; SFTP: secure file transfer protocol; WORDS: Women and Oncologists Reaching Decisions about Surgery.

As with many large research projects, attempting to explore the larger project process through deconstructive processes (ie, considering each module separately) overlooks the big-picture interactions between the different work processes. Within our project, four principal subprocesses existed: the clinical research, the IRB, the app development, and informatics. The tight coupling of these four processes created conditions where an inability to complete one step became a rate-limiting factor for progress in other parts of the project, even in workflows that seemed unrelated (Figure 1). For example, changes in the data storage structure in the informatics workflow necessitated additional consultations with the IRB. The IRB process had to be completed before the clinical workflow could proceed. Likewise, difficulties with getting the research software installed on patient phones, due to a Wi-Fi configuration issue, required changes to the underlying software and a subsequent change to the informatics workflow.

## Conclusions

The use of mHealth tools for scaling up health care research brings together new stakeholder groups, including IT teams and patients, which creates new opportunities. Like any novel research method, the use of mHealth for data collection carries a unique set of challenges for PIs who are used to traditional clinical research. The differences in pacing and work tempos between clinical research and software-driven, patient-participatory research are initially unexpected and have to be adjusted for. When combined with the variable pacing of software development, researchers should be prepared to devote additional time and energy to coordinating both the internal and external members of the research team. This time and energy will likely prove to be good investments, as mHealth techniques will likely gain prominence in the research community since they allow the clinical research community to collect valuable data in new ways. Unfortunately, the same unique qualities that allow mHealth technology to collect new types of data require adjustment in supporting infrastructures as well. While some institutions have pioneered new methods of supporting this work, many seem either unaware of new needs or unable to provide mHealth research support. Ultimately, mHealth will benefit clinical research by providing us the opportunities to gain insights into how patients actually live their lives, allowing us to create treatment strategies that work with, not against, patient lifestyles.

## Abbreviations

AWS: Amazon Web Services
DMZ: demilitarized zone
IRB: Institutional Review Board
IT: information technology
mHealth: mobile health
PHI: protected health information
PI: principal investigator
SFTP: secure file transfer protocol
WORDS: Women and Oncologists Reaching Decisions about Surgery
